# Development of the gut microbiome in early life

**DOI:** 10.1113/EP089919

**Published:** 2022-01-30

**Authors:** Sinead Ahearn‐Ford, Janet E. Berrington, Christopher J. Stewart

**Affiliations:** ^1^ Clinical and Translational Research Institute Newcastle University Newcastle upon Tyne UK; ^2^ Newcastle Neonatal Service Newcastle Hospitals NHS Trust Newcastle upon Tyne UK

**Keywords:** early life, gut microbiome, microbiota, organoids, preterm infant

## Abstract

**New Findings:**

- **What is the topic of this review?**
  The importance of the early life gut microbiome, with a focus on preterm infants and microbially related diseases. Current techniques to study the preterm gut microbiome are appraised, and the potential of recent methodological advancements is discussed.
- **What advances does it highlight?**
  Recent findings in the field achieved by the application of advanced technologies, the applicability of intestinally derived organoid models to study host–microbiome interactions in the preterm gut, and recent developments in enhancing the physiological relevance of such models. Preterm intestinally derived organoids may provide novel insights into the mechanisms underlying preterm disease, as well as diagnosis and treatment opportunities. These models have huge translational potential, offering a step towards precision medicine.

**Abstract:**

Accumulating evidence affirms the importance of the gut microbiome in both health and disease. In early life, there exists a critical period in which the composition of gut microbes is particularly malleable and subject to a wide range of influencing factors. Disturbances to microbial communities during this time may be beneficial or detrimental to short and long‐term health outcomes. For infants born prematurely, naïve immune systems, immature gastrointestinal tracts and additional clinical needs put this population at high risk of abnormal microbial colonisation, resulting in increased susceptibility to diseases including necrotising enterocolitis (NEC) and late‐onset sepsis (LOS). Traditional cell culture methods, gnotobiotic animals, molecular sequencing techniques (16S rRNA gene sequencing and metagenomics) and advanced ‘omics’ technologies (transcriptomics, proteomics and metabolomics) have been fundamental in exploring the associations between diet, gut microbes, microbial functions and disease. Despite significant investment and ongoing research efforts, prevention and treatment strategies in NEC and LOS remain limited. Recent endeavours have focused on searching for new, more physiologically relevant models to simulate the preterm intestine. Preterm intestinally derived organoids represent a promising in vitro approach in the study of host–microbiome interactions in the preterm infant gut, offering new and exciting possibilities in this field.

## 1 HISTORY

While using a self‐designed, handmade microscope to inspect his own stool, Antonie van Leeuwenhoek made the earliest observation of ‘animalcules’ (Leeuwenhoek, 1681). Since then, numerous approaches have been developed and refined to help study the trillions of tiny microscopic creatures that colonise the human body. Today, advances in cell culture, gnotobiotic animals, sequencing methods and powerful ‘omics’ technologies (Beck et al., 2021) have been indispensable in deepening our understanding of the complex human microbiome, that is, the community of microorganisms (bacteria, protozoa, fungi, viruses and bacteriophages) inhabiting the human body. Accompanying this methodological progress was the recognition of the importance of the microbiome, in particular the bacteria that reside in the gut, in both health and disease. Pivotal work leading to this realisation was conducted in gnotobiotic animals, discovering that (1) germ‐free animals have altered anatomical and physiological features and differ in their susceptibility to disease; (2) some of these features may be restored by bacterial colonisation; and (3) disease phenotypes can be transferred from sick animals or humans to healthy hosts by transplanting gut microbial communities (Kostic et al., 2013). Further, the clinical usefulness of manipulating the gut microbiome was realised in 1958, when four patients were successfully treated for the relief of intestinal inflammation by the transplantation of stool from healthy donors (Eiseman et al., 1958). Now, it is widely accepted that the gut microbiota play fundamental roles in defining the physiology of the host throughout the life course, developing and modulating the immune system, host metabolism and protection from infectious species. Nevertheless, these microbes have also been directly and indirectly implicated in the pathogenesis of multiple diverse disease states (Sommer & Bäckhed, 2013).

## 2 MICROBIOME SEQUENCING

Advances in high‐throughput nucleotide sequencing technologies have revolutionised gut microbiome research, enabling deep investigations into how bacterial communities develop over time, the factors that influence developing communities, and their links to health and disease. The most common approach to profile the microbiome is 16S rRNA gene sequencing, which amplifies this universal gene in bacteria. This relatively cost‐effective method typically provides resolution to genus level and is especially powerful for low‐biomass samples. While 16S rRNA gene sequencing has routinely been employed over the past decades, shotgun metagenomic sequencing has become increasingly popular in recent years. In contrast to 16S rRNA gene sequencing, in which a specific bacterial gene region is targeted, metagenomic sequencing sequences the entirety of a sample. This is more expensive, but will detect non‐bacterial microbes (e.g., fungi, viruses and bacteriophages), allows identification to species and strain level, and provides information on the functional genetic capacity of the microbes (Ranjan et al., 2016).

## 3 GUT MICROBIOME IN INFANCY

### 3.1 Term infants

**FIGURE 1:**
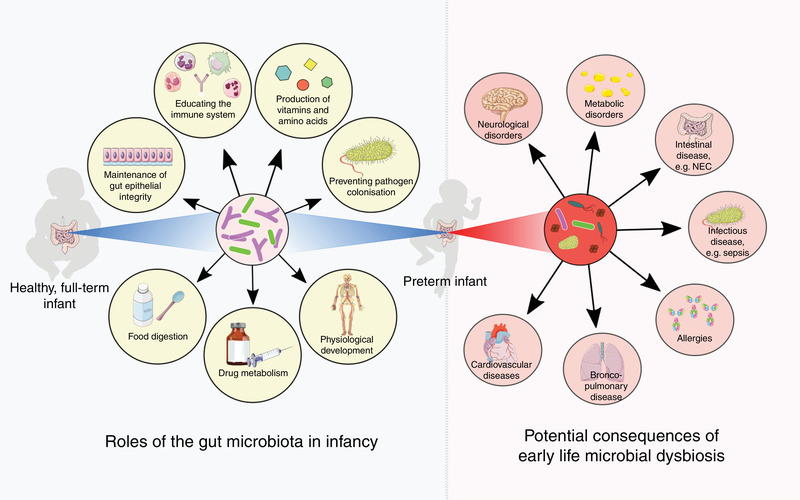
Roles of the infant gut microbiota (left) and potential short‐ and long‐term consequences of early life microbial dysbiosis (right), which may be common in preterm infants. NEC, necrotising enterocolitis. Image was created using Servier Medical Art (http://smart.servier.com/)

The gut microbiome plays essential roles in human health, especially during infancy (Figure 1). The first year of life is considered a crucial window of opportunity to manipulate the microbiome and train the immune system, to promote short and much longer‐term health outcomes. Thus, bacteria living in the neonatal gut have been a focal point of microbiome research. The first extensive exposure to these organisms occurs at birth: soon after an infant exits the womb, microbes begin to enter the human body and in time establish themselves in the gut (Milani et al., 2017).

Studies utilising molecular technologies have described neonatal gut microbiota development in detail. The TEDDY study took place across six research centres in Europe and the USA, and collected 12,500 stool samples from over 900 term infants between 3 and 46 months of life (Stewart et al., 2018). Using both 16S rRNA and metagenomic sequencing, the study defined three distinct phases of microbiome progression over the first 3 years of life, before the microbiome stabilises into an adult‐like configuration. The TEDDY study further demonstrated the association between term infant gut microbiome structure and breastfeeding, mode of delivery, geographical location, and living with siblings and/or furry pets (Stewart et al., 2018), while other studies are similarly applying these technologies to showcase the impact of maternal factors (Chu et al., 2016; Ferretti et al., 2018), antibiotic treatments (Yassour et al., 2016), host genetics (Goodrich et al., 2014), diet and ethnicity (Stearns et al., 2017), assisted reproductive technology (Lu et al., 2020), and potentially even the SARS‐CoV‐2 pandemic (Romano‐Keeler et al., 2021). Perturbations to the gut ecosystem during this critical period of development can prove detrimental; drastic shifts in neonatal community structure, often referred to as ‘dysbiosis’ (a term considered problematic by some, e.g., Hooks & O'Malley, 2017), have been linked to a number of disease pathogeneses. An increasing number of animal and in vitro studies are exploring the functional impacts of an altered gut microbial community, and findings are being corroborated by epidemiological and molecular studies. As such, early life imbalances in the gut microbiota have been associated with acute gastrointestinal and bacterial diseases, as well as allergic diseases, atopic dermatitis, asthma, irritable bowel syndrome, obesity, diabetes, cardiovascular diseases and a range of neurological disorders, among others, in later life (Bejaoui & Poulsen, 2020; Milani et al., 2017; Sandall et al., 2018).

### 3.2 Preterm infants

**FIGURE 2:**
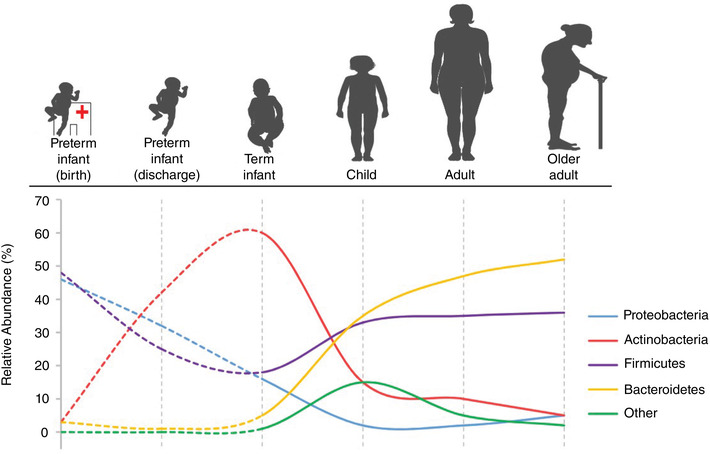
Development of the gut microbiota throughout life. Relative abundances of bacterial phyla (Proteobacteria, Actinobacteria, Firmicutes, Bacteroidetes and other) are depicted for preterm infants (at birth and at discharge from hospital), term infants, children, adults and older adults. Infants’ relative abundances were derived from work by (Masi et al., 2021; Stewart et al., 2018; Vaiserman et al., 2017). Image was created using Servier Medical Art (http://smart.servier.com/)

Gestational age at birth is another highly influential factor in shaping the gut microbiome in infancy (La Rosa et al., 2014). In the case of the premature infant, an additional, entirely unique set of factors governs the gut ecosystem. Before making contact with the outside world, preterm infants are more likely to be exposed to microbes via premature rupture of membranes and intra‐amniotic infection (Tchirikov et al., 2018). Following birth, the preterm infant is cared for within the neonatal intensive care unit (NICU) environment, with altered exposure to microbes, involving an increased exposure to hospital‐acquired bacteria and reduced access to parental/family bacteria. In addition, they undergo clinical care that is distinct from that of healthy term infants, including specific feeding practices, receipt of antibiotics, ventilation and intravenous line placement. Such factors are known to impact the developing gut microbiome in preterm infants (Jia et al., 2020; Yap et al., 2021; Zwittink et al., 2017). Moreover, preterm infants have immature gastrointestinal tracts and naïve immune systems (Henderickx et al., 2019). A study using a gnotobiotic mouse model has shown that the premature microbiota may actually further affect intestinal development, influencing features such as villus height and crypt depth, intestinal barrier integrity, and cell proliferation and differentiation (Yu et al., 2016). With this early start to life, preterm infants are known to differ in microbial diversity, pace of colonisation, and the specific bacteria that colonise the gut, when compared to term infants (Jia et al., 2020; La Rosa et al., 2014; Yap et al., 2021) (Figure 2). For premature infants, these perturbations often result in higher risks of complications and disease (Henderickx et al., 2019) (Figure 1).

#### 3.2.1 Disease in preterm infants

The high susceptibility of preterm infants to early gut microbial dysbiosis, along with a predisposition to pathobiont colonisation due to the NICU environment and antibiotic use (Jia et al., 2020; Yap et al., 2021; Zwittink et al., 2017), contributes to an increased risk of developing both necrotising enterocolitis (NEC) and late‐onset sepsis (LOS). NEC is still poorly understood, but in many cases is likely an immune‐mediated bowel disease. Between 2% and 7% of preterm infants (born <32 weeks’ gestation) develop NEC (Battersby et al., 2018) and the disease is a leading cause of morbidity and mortality in this population. LOS similarly contributes to a large proportion of preterm deaths and affects up to 36% of premature neonates (Dong & Speer, 2015). As well as translocation through the skin, less effective tight junctions contribute to ‘leakiness’ of the preterm intestinal epithelium and may result in LOS in which the causative agent is an enteric microbe. Many factors associated with microbiome perturbations have been linked to both NEC and LOS, including antibiotic administration (overuse: Esmaeilizand et al., 2018; or underuse: Li et al., 2020), medical interventions and feeding mode (Dong & Speer, 2015; Niño et al., 2016). LOS, by definition, is a microbially related condition, and in recent years the medical community has investigated and defined the relevance of the microbiome in NEC. Still, both diseases represent a serious burden to premature infants, and the exact pathophysiology of NEC remains elusive.

Substantial efforts have been made over the past few decades to define the microbial characteristics of NEC and LOS, and compelling data have associated both conditions with intestinal dysbiosis. Both culture‐dependent and culture‐independent methods have demonstrated differences in the microbiota of preterm infants that develop NEC, compared to matched healthy controls. In a study combining metagenomics and metabolomics (the study of metabolites), researchers have demonstrated instability of the gut microbiome in NEC infants prior to onset of disease, and also identified metabolites associated with NEC diagnosis (Stewart et al., 2016), offering the opportunity for development of biomarkers. Although no uniform microbial signature has been found in NEC, numerous recent investigations have described an abnormal intestinal ecosystem in comparison to healthy preterm infants, and some have associated particular bacterial taxa with NEC (Niño et al., 2016; Stewart et al., 2012, 2016; Ward et al., 2016). However, findings are inconsistent and no causative bacterial strain has been found. A multi‐omic approach combining metagenomics and metabolomics has similarly been applied in the investigation of LOS, finding alterations in the gut microbiome in this population (Stewart et al., 2017). Other studies have corroborated the finding of a distortion in normal microbiota composition preceding disease onset (El Manouni El Hassani et al., 2021; Graspeuntner et al., 2019; Mai et al., 2013; Stewart et al., 2012).

Human milk has a protective role against both NEC and LOS (Dong & Speer, 2015; Niño et al., 2016) and is the single most important factor in shaping the term infant gut microbiome (Stewart et al., 2018). Human milk is the recommended source of nutrition for neonates; it confers immunological benefits to the infant and provides nutrients to specialised microbes that similarly benefit the infant's immune system (Henrick et al., 2021; Masi et al., 2021; Stewart, 2021). Specifically, breastfeeding induces an early life abundance of the bacterial genus *Bifidobacterium* (Stewart et al., 2018). Human milk oligosaccharides are complex sugars found in abundance in human milk. They provide selective nutritional advantages to Bifidobacteria and largely account for the significant proportion of *Bifidobacterium* found in the healthy infant gut (Garrido et al., 2013). Bifidobacteria have been shown to possess immunomodulatory functions, protect from pathogens and improve barrier function (Milani et al., 2017). Moreover, a lack of Bifidobacteria in infancy has been linked to systemic inflammation and immune dysregulation (Henrick et al., 2021). Importantly, preterm babies with NEC (Masi et al., 2021; Stewart et al., 2016) and LOS (Mai et al., 2013) have relatively decreased abundances of *Bifidobacterium* spp., and in the case of NEC, supplementation with probiotics containing Bifidobacteria, among other species, may reduce the risk of disease (van den Akker et al., 2020). In addition, recent data suggest that human milk oligosaccharide profiling may be useful in predicting NEC onset (Masi et al., 2021).

## 4 MOVING BEYOND MICROBIOME ASSOCIATIONS

Molecular methods have enabled the rapid and comprehensive identification of microbial species related to disease, which has opened up new research possibilities in the therapeutic efforts against preterm NEC and LOS. Still, metagenomic sequencing of non‐invasive stool samples may fail to accurately capture the community functioning of the microbiome within the different compartments of the intestine. Transcriptomics, proteomics and metabolomics (the study of gene expression, proteins and metabolites, respectively) can assist with understanding this functional capacity (Beck et al., 2021; Renwick & Stewart, 2021). Recent work has integrated these multi‐omic techniques to substantially improve our understanding of host–microbiome interactions (Graspeuntner et al., 2019; Stewart et al., 2017, 2016, 2018; Yap et al., 2021; Zwittink et al., 2017). Nevertheless, such work typically yields associations and often more questions than answers. Thus, to build from this work and to understand potential mechanisms, a representative model of the preterm intestinal gut environment is essential.

One such model is the preterm intestinally derived organoid. Intestinal organoids are self‐organising three‐dimensional epithelial structures that simulate and retain some of the complex structural and functional properties of the native intestinal microenvironment. Intestinal organoids are derived directly from a human source (intestinal biopsy/resected samples) and enable prolonged culture and assessment of the intestinal epithelium. As the cultures are patient‐derived, these models also retain unique genetic and epigenetic features of individuals. Importantly, life stage was also recently shown to be captured in intestinal organoids, where lines derived from preterm infants and adults differed significantly in their gene expression profiles (Masi, Fofanova, et al., 2021). This difference was further emphasised when culturing the organoids with viable bacteria. Thus, co‐culture with microbes specific to the patient further increases the relevance of this model.

Although intestinal organoids have not yet been integrated in the study of diseases of preterm infants, recent work has elucidated an effective protocol to produce preterm intestinally derived organoids from intestinal stem cells isolated from surgically resected tissue (Stewart et al., 2020). Other work has developed an experimental enteroid model of NEC by inducing an inflammatory response with lipopolysaccharide, based on prior knowledge of experimental NEC in cell culture and animal models (Ares et al., 2019). Approaches have also sought to make such models more physiologically relevant, including anaerobic co‐culture and microfluidic ‘organ‐on‐a‐chip’ (Bein et al., 2018; Fofanova et al., 2019). Moreover, recent work involving the exposure of preterm intestinal cell cultures to the infant's faecal filtrates was able to connect specific bacterial phyla to inflammatory mediators, in a method that could also be applied to organoids (Gibbons et al., 2021). These recent developments undoubtedly have potential to benefit the study of host–microbiome interaction in preterm disease, including mechanisms and potential interventional strategies. Beyond research, the use of such relevant, personalised models could have huge translational potential: evidence can be directly brought back to clinicians to facilitate future informed, personalised decisions in healthcare.

## 5 CONCLUDING REMARKS

With improved neonatal care practices, the number of surviving preterm infants is increasing, along with subsequent increases in the rates of NEC and LOS. Still, treatment strategies prove inadequate and prevention appears out of reach. As an easily manipulated and thus incredibly attractive target, the gut microbiome and the tools used to explore it are now more important than ever. Preterm intestinally derived organoids offer promise as a novel strategy to investigate host–microbiome interactions in the preterm gut. Further, due to advances in enhancing the physiological relevance of these models as well as the personalised nature of patient‐derived organoids, preterm intestinally derived organoids may have a significant impact on future clinical work. Gaining a deeper understanding of the microbiome using relevant methodology will enable the exploration of new targeted therapeutic strategies in preterm disease as well as in a range of other pathologies.

## COMPETING INTERESTS

C.J.S. and J.E.B. declare receiving lecture honoraria from Danone Early Life Nutrition and Nestle Nutrition Institute, but have no share options or other conflicts.

## AUTHOR CONTRIBUTIONS

S.A.F. drafted the initial manuscript and produced the images. J.E.B. and C.J.S. contributed to critical revisions. All authors have read and approved the final version of this manuscript and agree to be accountable for all aspects of the work in ensuring that questions related to the accuracy or integrity of any part of the work are appropriately investigated and resolved. All persons designated as authors qualify for authorship, and all those who qualify for authorship are listed.
